# T-Cell Immunity to Influenza in Older Adults: A Pathophysiological Framework for Development of More Effective Vaccines

**DOI:** 10.3389/fimmu.2016.00041

**Published:** 2016-02-25

**Authors:** Janet E. McElhaney, George A. Kuchel, Xin Zhou, Susan L. Swain, Laura Haynes

**Affiliations:** ^1^Health Sciences North Research Institute, Sudbury, ON, Canada; ^2^UConn Center on Aging, University of Connecticut School of Medicine, Farmington, CT, USA; ^3^Department of Immunology, University of Connecticut School of Medicine, Farmington, CT, USA; ^4^Department of Pathology, University of Massachusetts Medical School, North Worcester, MA, USA

**Keywords:** influenza, vaccination, immunosenescence, antibody, cell-mediated immunity

## Abstract

One of the most profound public health consequences of immune senescence is reflected in an increased susceptibility to influenza and other acute respiratory illnesses, as well as a loss of influenza vaccine effectiveness in older people. Common medical conditions and mental and psychosocial health issues as well as degree of frailty and functional dependence accelerate changes associated with immune senescence. All contribute to the increased risk for complications of influenza infection, including pneumonias, heart diseases, and strokes that lead to hospitalization, disability, and death in the over 65 population. Changes in mucosal barrier mechanisms and both innate and adaptive immune functions converge in the reduced response to influenza infection, and lead to a loss of antibody-mediated protection against influenza with age. The interactions of immune senescence and reduced adaptive immune responses, persistent cytomegalovirus infection, inflammaging (chronic elevation of inflammatory cytokines), and dysregulated cytokine production, pose major challenges to the development of vaccines designed to improve T-cell-mediated immunity. In older adults, the goal of vaccination is more realistically targeted to providing clinical protection against disease rather than to inducing sterilizing immunity to infection. Standard assays of antibody titers correlate with protection against influenza illness but do not detect important changes in cellular immune mechanisms that correlate with vaccine-mediated protection against influenza in older people. This article will discuss: (i) the burden of influenza in older adults and how this relates to changes in T-cell function, (ii) age-related changes in different T-cell subsets and immunologic targets for improved influenza vaccine efficacy in older, and (iii) the development of correlates of clinical protection against influenza disease to expedite the process of new vaccine development for the 65 and older population. Ultimately, these efforts will address the public health need for improved protection against influenza in older adults and “vaccine preventable disability.”

## Burden of Influenza, Immune Senescence, and Loss of Vaccine Effectiveness

### Impact of Influenza in Older Adults

Aging is associated with a decline in cell-mediated immunity and a dramatic increase in the morbidity and mortality from community-acquired pneumonia, particularly in the population age 75 years and older (1). Influenza is the most common cause of viral pneumonia in older adults (1) and associated complications, including ischemic heart disease, cerebrovascular events, and diabetes in adults age 70 years and older during annual influenza epidemics, suggest that influenza illness is the major cause of excess mortality in this population (2). Rising hospitalization rates, increased lengths of hospital stay, and 36,000 deaths annually in the U.S. during the influenza season in the over 65 population despite widespread vaccination programs (3–5) are increasingly raising concerns about influenza vaccine effectiveness. Moreover, yearly immunization fails to induce immunity to new strains of influenza, leaving the population susceptible to pandemic outbreaks.

### Contribution of Multi-Morbidity to Risk for Influenza and Related Disability

**Figure 1:**
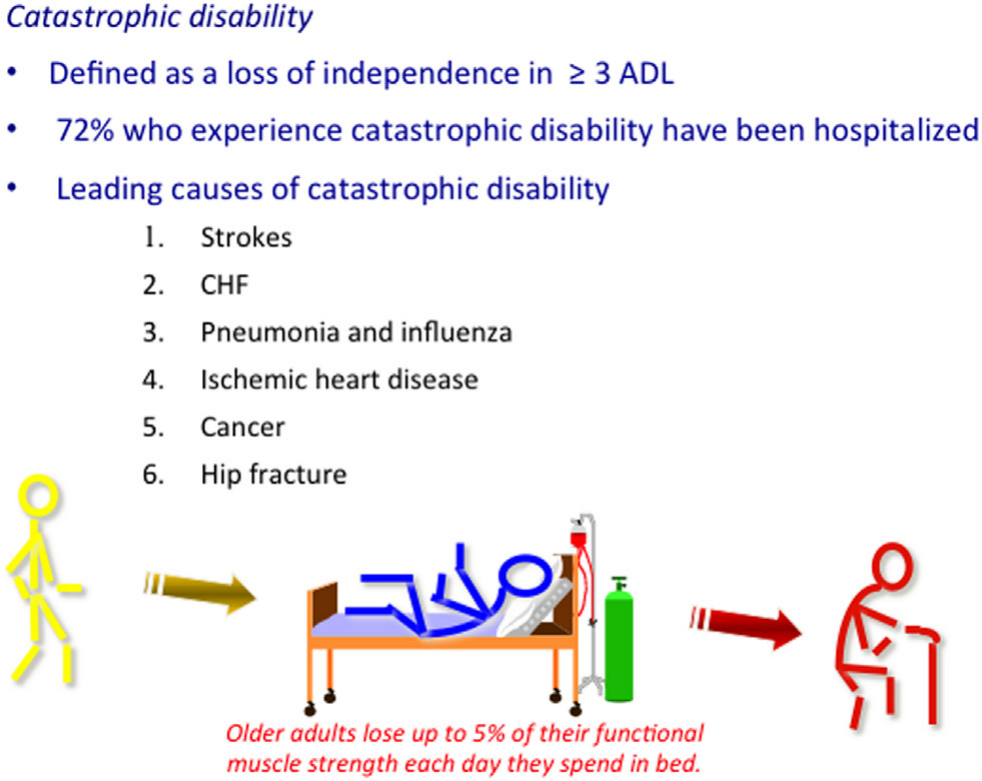
**Multi-morbidity that is defined as the presence of two or more chronic conditions affects 50% of older people, most of whom enjoy an active life in the absence of an acute illness or injury (~70% of the over 65 population as represented by yellow stick figure)**. Influenza has been associated with the six leading causes of catastrophic disability. It has been postulated that the inflammatory response to the influenza virus leads to these serious complications, which are common causes of hospitalization in older adults. During periods of inactivity, older adults lose up to 5% of their functional muscle strength every day they are in bed and may never recover, thus, being identified as a frail older adult (represented by the red stick figure) at hospital discharge. Influenza vaccines that improve protection against influenza illness and related complications provide a significant public health opportunity to promote health aging and vaccine preventable disability.

Multi-morbidity that is defined as the presence of two or more chronic conditions occurs in two-thirds of population age 65–84 years old and over 80% of those age 85 years and older (6). The intersection between multi-morbidity and outcomes of influenza illness is obvious when one considers that the risk for hospitalization and death due to influenza illness in older adults increases with age, chronic disease burden, prior hospitalization for pneumonia, and co-morbidities, including lung, heart and renal diseases, dementia, strokes, and hematological and non-hematological malignancies (7). Hospitalization in older adults confers a 60-fold increased risk for disability but the link between influenza and disability and frailty in older adults is only beginning to be understood (8, 9). The six leading causes of catastrophic disability (defined as the loss of independence in three or more basic activities of daily living), including strokes, congestive heart failure, pneumonia, ischemic heart disease, cancer, and hip fracture (10), have all been linked to influenza (Figure 1) but this type of data is not captured in the typical databases used to estimate influenza vaccine effectiveness. Rates of long-term morbidity and disability following influenza illness in older people are predictable and will increase in parallel with hospitalization rates, impacting not only on cost to the health care system but also on the quality of life of older persons.

### Decline in Influenza Vaccine Effectiveness in Older Adults

Recent studies have questioned the effectiveness of current influenza vaccines in older adults, which are composed of inactivated virus or subunit proteins and have used the same egg-based technology for over 50 years. Govaert et al. conducted the only published randomized, placebo-controlled trial of influenza vaccination in older adults, providing an estimate of vaccine efficacy of only 50% in a relatively healthy cohort (11). Influenza vaccination has since become a standard of care and it is now considered unethical to conduct placebo-controlled trials. Thus, current estimates of vaccine effectiveness are mainly derived from observational studies comparing vaccinated and unvaccinated older adults. Varying degrees of specificity for identifying influenza illness and related unmeasured confounding (12), lack of documentation of prior vaccination history, and only rudimentary methods for measuring the combined effects of chronic disease burden, functional status, and frailty contribute to confounding in the analysis of vaccine effectiveness. As an example, older adults with chronic diseases and related disability would be expected to have more frequent health care contacts and be more likely to receive annual influenza vaccination. Annual repeated vaccination contributes to improved vaccine efficacy, while the presence of co-morbidity is associated with a reduction in influenza vaccine effectiveness (11, 13–15); these two opposing effects become important variables in establishing estimates of vaccine effectiveness. In fact, a recent observational study of hospitalized adults with laboratory-confirmed influenza illness conducted by the Canadian PCIRN Serious Outcomes Surveillance network (16) showed similar estimates of influenza vaccine effectiveness in young and older adults after adjustment for the combined effects of chronic disease burden, functional status, and frailty using the Frailty Index (17, 18). In other words, vaccinated individuals were more frail than unvaccinated older adults in this hospital cohort, which counters the argument for “healthy vaccinee bias” leading to overestimates of vaccine effectiveness in the over 65 population (19, 20). Furthermore, a Cochrane review (21) questioning the effectiveness of influenza vaccine in older adults has been challenged on the basis of over-stratification of the data and loss of statistical power, and lack of separation of data from good vs. poor match vaccine to the circulating strains of influenza virus, and years with low vs. high levels of circulation of influenza. When these factors were taken into consideration, a re-analysis of the same studies included in the Cochrane review, provided estimates of vaccine effectiveness of 30% in older adults, consistent with the existing literature (22, 23). Given that influenza vaccination remains a cost-saving medical intervention in older adults in spite of the well-recognized decline in vaccine effectiveness in older adults, recent advances in vaccine technology offer an unprecedented opportunity to improve influenza vaccines for this population. However, a more complete understanding of the impact of immune senescence – the changes that occur with aging – and the complexity and heterogeneity of the immune response to influenza in older adults is needed.

### Interactions between Inflammaging, Frailty, and Immune Senescence

Understanding how genetics and exposures to a host of factors that influence gene regulation and protein expression, determine longevity, and predict the health trajectory with aging have led to the concept of “inflammaging,” the adverse changes associated with aging and increased serum levels of inflammatory cytokines (24, 25). Chronic inflammation appears to drive much more than immune senescence leading to increased susceptibility to infectious diseases and now also appears to be fundamentally implicated in “unsuccessful” aging (25) manifested as increasing frailty with age.

Frailty as a syndrome encompasses a person’s chronic medical conditions, functional status, and risk of mortality (26). It is associated with a loss of physiological reserve and ability to resist environmental stressors, and increased risk of functional decline (27, 28). Through inflammatory mechanisms, chronic diseases contribute to declining immune function and cause organ-specific changes in susceptibility to pathogens, particularly in the lungs, thus, acting synergistically to impair mucosal barrier function and innate and adaptive immunological defense mechanisms. Consequently, protection against influenza or serious complications thereof depends not only on the ability of vaccines to reverse age-related changes in adaptive immune function but also overcome the loss of the mucosal barrier function of the lungs and innate immune mechanisms that may be altered by chronic disease processes, inflammation, and frailty. The Frailty Index based on both clinical and laboratory markers is emerging as a novel method for incorporating these variables into a measure of overall health status and stratifying risk for serious complications, including disability from influenza illness (16, 29).

In the evaluation of the immune response to influenza vaccination, frailty must be considered as a multifactorial syndrome that represents a reduction in physiological reserve and in the ability to resist environmental stressors (27, 28, 30). Frailty is generally recognized to be age-associated and common in older adults, and an important factor in the risk for complications of influenza illness. While functional dependence (e.g., not being able to bath independently) has been identified a potential confounder in the analysis of influenza vaccine effectiveness in the over 65 population (20), the impact of level of frailty on the response to influenza vaccination has yet to be studied. Frailty is complex and dynamic in nature, and reflects the loss of adaptive capacity of the organism. Frailty as a syndrome in the geriatric population encompasses a person’s chronic medical conditions, functional status, and risk of mortality (26). The Frailty Index, which measures the degree to which a person is frail, accounts for more than just physical frailty (31), relates to the accumulation of deficits in all aspects of self-reported health and functional status (32), is a more sensitive measure of degree of frailty, and predicts mortality risk (33). Increased frailty due to influenza illness has been demonstrated using a 40-item Frailty Index where older adults with influenza had, on average, three more deficits at hospital discharge compared to their baseline (34).

### Role of Cytomegalovirus in T-Cell Responses to Influenza

Persistent cytomegalovirus (CMV) infection has also been linked to functional decline in older adults (35) and it causes T-cell senescence (36), and thereby contributes to the age-associated changes in immune function. T-cell responses in the aged are very different from those in the young. Thymic involution and a decline in naïve T-cell output with increasing age, together with a lifetime of exposure to a variety of pathogens, lead to a dramatic reduction in the naïve T-cell pool and a relative increase in the proportion of memory T cells. Moreover, the remaining naive CD4 T cells make poor helper responses, undermining the ability of vaccines to protect against new pathogens or variants. Within the total memory pool, arguably, the most dramatic functional changes occur in the CD8+ T-cell subset, where increased proportions of CD8^+^CD28^−^ T cells have been associated with poor antibody responses to influenza vaccination (37, 38), and seropositivity for CMV (39). Indeed, it has been shown that most of these CD8^+^CD28^−^ memory T cells are part of large clonal expansions that are specific for persistent viruses, mainly CMV (40). However, a direct link between changes in CD8^+^ T cells related to CMV seropositivity, and the dramatic increase with age in the risk for complicated influenza illness has yet to be made. Given that at least 90% of those 80 years and older are CMV seropositive (41) and that persistent CMV infection drives T cells to a late-differentiated state associated with loss of function, CMV may have a significant role in cell-mediated protection against viral illnesses, such as influenza.

The systemic upregulation of the inflammatory cytokine, interleukin-6 (IL-6), in association with high CMV antibody titers has been associated with increased frailty (42). Recently, studies in mice have shown that in responses to antigens that are not associated with high levels of pathogen-associated danger signals, such as most unadjuvanted vaccines, IL-6 plays a critical role in generation of T follicular helper CD4 (Tfh) cells that are essential for optimum generation of B-cell antibody response and memory (43). However, the IL-6 that is needed is not systemic IL-6, but comes from the dendritic cells (DC) presenting antigen to CD4 T cells (44, 45). Thus, while the elevated systemic IL-6 associated with inflammaging is deleterious, IL-6 production by DC is critical to optimum immunity.

Anti-inflammatory cytokines, such as IL-10, can counterbalance the negative effect of elevated systemic IL-6, and have been associated with successful aging, but excess IL-10 can also result in a diminished resistance to infectious diseases and a decline in the antibody response to influenza vaccination (46). Increased IL-10 production in macrophages following vaccination in older adults predicts a poor antibody response (46), and is associated with a decline in the IFNγ:IL-10 ratio in response to influenza virus challenge with aging (47). Lower IFNγ:IL-10 ratios and levels of the cytolytic mediator, granzyme B, in influenza-challenged peripheral blood mononuclear cells (PBMC) correlate with increased risk of influenza in vaccinated older adults (48). These results are based on outcomes of influenza A/H3N2 illness, the most common subtype affecting older adults (49, 50), and has the greatest impact in terms of hospitalization and death in older adults (5). Thus, A/H3N2 strains should be the primary focus for developing novel correlates of clinical protection against influenza in this population.

### Limitations of Antibody-Mediated Protection from Influenza in Older Adults

Current influenza vaccines are standardized by the amount of hemagglutinin (HA) contained in the vaccine, and changes in HA inhibition (HAI) antibody titers in response to influenza vaccination are the industry standard for measuring vaccine efficacy. Thus, influenza vaccines are designed to provide antibody-mediated protection against infection or “sterilizing immunity” mainly by influenza strain-specific antibodies against the surface glycoproteins, HA, and neuraminidase (NA). However, the diminished mechanical barrier function in the lungs (51), a decline in innate immune mechanisms (52), and changes in the quality and quantity of HAI antibodies with age (53), may also contribute to higher rates of infection of airway epithelial cells in older adults. Due to the strain specificity of the HAI antibody response, the degree of mismatch of the vaccine strains to the circulating strain of influenza virus correlates with a significant reduction in vaccine efficacy in all age groups.

### Age-Related Changes in T-Cell Subsets May Affect Protection from Influenza in Older Adults

**Figure 2:**
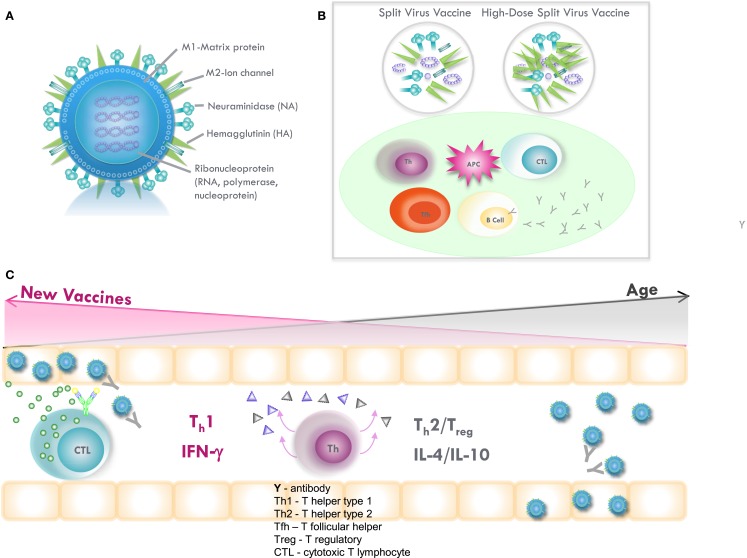
**(A)** Influenza virus contains the surface glycoproteins, hemagglutinin (HA), and neuraminidase (NA), which are strain specific in directing the antibody response to influenza vaccination. The internal proteins, including nucleoprotein (NP) and matrix (M1) protein, are conserved across the different subtypes of influenza A and, thus, confer cross-protection. Subunit vaccines contain the surface glycoproteins, while split-virus vaccines contain both surface glycoproteins and internal proteins of the virus. High-dose, split-virus (HD) vaccines are designed to improve antigen presentation from antigen-presenting cells (APCs) in the lymph node, eliciting greater antigen-specific antibody responses and potentially improving the cytotoxic T lymphocyte response for improved viral clearance from the lungs. **(B)** Inflammation stimulated by the injection and potentially enhanced by adjuvants, such as MF59 or increased amounts of vaccine antigen (HD vaccine), facilitates the activation of the APC and uptake of inactivated virus. The APC then migrates to the local lymph node to interact with B cells, CD4^+^ T helper (Th) and T follicular helper (Tfh) cells, and CD8^+^ cytotoxic T lymphocytes (CTL). APCs present vaccine-derived peptides on MHC II stimulating Tfh and the production of strain-specific antibodies by B cells, the cross-reactivity of which is enhanced by vaccine adjuvants, such as MF59. Memory CD8^+^ T cells from prior exposure to natural influenza infection can be restimulated by vaccination, and may be enhanced by HD vaccines containing internal proteins. **(C)** Influenza infection in the lungs activates Th_1_/Th_2_/T_reg_ in the adjacent lymph nodes and stimulates a T_h_1 response with IFNγ production to effectively activate memory CTL, which clear virus from the lungs. However, age-related changes drive a Th_2_/T_reg_ response to infection, and IL-10 production suppresses the CTL response. A shift toward a protective T_h_1 and CTL response to infection may be stimulated by increasing the amount of internal proteins and/or the use of adjuvants in vaccines targeted to improve protection against influenza in older adults.

Vaccination also stimulates influenza-specific CD4 and CD8 T-cell memory cells that generate effectors that are recruited to the lungs to clear virus-infected cells in the lungs (Figure 2), thus, providing “clinical protection” against disease. T-cell protection becomes increasingly important as we age. Influenza-specific memory T cells are highly cross-reactive; CD4 and CD8 epitopes are specific for the conserved protein sequences across different influenza strains and, thus, do not depend on an exact match of the vaccine strain with the circulating strain of influenza virus. During influenza infection, many subsets of CD4 and CD8 effectors are generated that contribute to influenza virus clearance. In mice, CD4 effectors of multiple subsets including helper type 1 (T_h_1) cells producing interleukin-2 (IL-2) and IFNγ that recruit virus-specific cytotoxic T lymphocytes (CTL) to the lungs to kill influenza-infected lung epithelial cells (54). Also, Tfh effectors that are necessary to help B cells become neutralizing Ab-producing cells play critical, often synergistic roles in combating viruses (55). Other Th cells, especially regulatory T cells (Treg) can produce high levels of IL-10 at the site of infection and delay recovery (56). IL-10 levels increase with aging in response to vaccination (46) and in the subsequent response to influenza virus challenge (57) but multiple sources of IL-10 production, including macrophages, B cells, and Treg, have now been identified (46, 58, 59) and need to be considered in modulating the T-cell response through vaccination.

### Memory T-Cell Responses to Influenza Vaccination in Older Adults

Vaccines can provide protection either by generating new responses from naive T and B cells or by boosting memory cells formed during previous exposure by vaccination or infection. In mice, it has been shown that memory T cells formed early in life retain function despite aging (60, 61). Humans have multiple encounters with influenza; so in most cases, the T cells involved in response to vaccine in elderly individuals will be memory T cells (Figure 2). Vaccination boosts memory T cells specific for both the surface glycoproteins and the internal proteins of the virus, although it has recently been shown that vaccination more effectively boosts CD4 T cells specific for the conserved internal proteins of influenza virus, matrix (M), and nucleoprotein (NP), in both young and older adults (62). Increased levels of IFNγ-producing CD4 (T_h_-type 1) and CD8 T cells (CTL) specific for the conserved internal proteins of influenza virus, matrix (M), and nucleoprotein (NP) correlate with protection against influenza in young adults (63, 64) corresponding to the key role of CTL in clearing influenza-infected cells from the lungs.

However, influenza-specific CTL activity and IFNγ production by memory T cells in response to influenza challenge decline with aging and are poorly stimulated by inactivated influenza vaccines (65, 66). Granzyme B (Grz B) is a key component of perforin-mediated killing by CTL, and low levels of GrzB activity generated in response to influenza challenge following vaccination predict increased risk (48, 67) and severity of (68) influenza illness in older adults. Given that the frequency of CD4 and CD8 T cells responding to NP or M protein-derived peptides correlate with cell-mediated protection, subunit vaccines containing only the surface glycoproteins (HA and NA) may not provide as potent a stimulus to T-cell memory. Indeed observational studies have shown a reduction in vaccine efficacy with subunit vaccines when compared to split-virus vaccines (containing both surface glycoproteins and internal proteins) in older adults (69). Since split-virus and subunit vaccines will generate equivalent antibody responses, the results of these observational studies highlight the importance of developing a correlate of T-cell-mediated protection to complement antibody titers as a surrogate of protection in the over 65 population.

### Persistent CMV Infection Affects the T-Cell Response to Influenza Vaccine

Cytomegalovirus seropositivity, inflammation, and level of frailty predict mortality in older adults (70), and are associated with loss of vaccine-mediated protection in older adults (71). We have found that CMV+ compared to CMV− older adults have abnormally increased levels of GrzB in resting T cells, which we have called “bGrzB” activity. This bGrzB activity is associated with an increase in the proportion of both CD4^+^ and CD8^+^ T cells that are GrzB^+^ and have a CD45RA^+^ late-differentiated T-cell phenotype or are CD28^−^ T cells (72). In the CD4 T-cell population, these CMV-specific late-differentiated T cells are associated with poor memory responses to the internal proteins (M and NP-derived peptides) of influenza virus (73). By contrast, CMV seropositivity has been associated with improved antibody responses to influenza vaccination in older adults, including those with well-controlled Type 2 diabetes (74). Others have shown that telomere length, independent of CMV serostatus, is a predictor of the robustness of the B-cell and CD8^+^ T-cell responses to influenza vaccination in older adults with longer telomere length, respectively, associated with improved antibody responses and increased frequency of M1-specific T cells (75). Our work has shown that late-differentiated T cells (CD45RA^+^GrzB^+^Perforin^−^) are particularly abundant in the CD8^+^ subset, with as many as 50% of these cells producing GrzB in the resting state, and are associated with poor CD8 T-cell cytolytic activity following influenza vaccination (76). Our unpublished studies have shown in a regression analysis that age, chronic disease, and CMV seropositive status are correlated with increased bGrzB activity (*R*^2^ = 0.278, *p* < 0.0001) and a poor response to influenza challenge (*R*^2^ = 0.375, *p* < 0.0001) in an *in vitro* model simulating the response to influenza vaccination (57). Thus, we propose bGrzB activity in resting T cells as a measure of the immunologic burden of terminally differentiated T cells in older adults.

## Opportunities for Targeting T Cells for Improved Protection with Influenza Vaccination

In considering how to improve influenza vaccines in the elderly, lessons learned in studies of mice could help us define what is missing in current strategies and where improvements might be made. We have shown that there is a key weakness that restricts aged naive CD4 T cells responses, namely that the initial antigen recognition step requires the DC presenting antigen to make high levels of IL-6 (44, 45) (Brahmakshatriya and Swain unpublished). Aged CD4 T cells appear to require more IL-6 to be triggered than young ones. In mouse models, the CD4 response can be enhanced by pre-activating the DC with agonists to toll-like receptors (TLRs) that elevate their potential for IL-6 production. Since systemic IL-6 is likely harmful, designing strategies to target both the antigen and agonist to DC could potentially provide a much better response of T cells, including the Tfh subset that is critical for helping the B-cell antibody response. Preliminary studies in aged mice indicate that introducing activated DC bearing T-cell influenza epitopes in addition to inactivated vaccine is sufficient to boost IgG antibody responses (Brahmakshatriya, unpublished).

### Age-Related Changes in T Follicular Helper Cells and Response to Influenza Vaccination

Overall, CD4 T-cell responses in aged individuals are not too dissimilar from those found in young adults following vaccination with an inactivated influenza vaccine. There is an initial expansion of responding CD4 T cells but this expansion is not sustained in older people, possibly due to lower levels of IL-7 (77). In order for a vaccine to induce high affinity, class-switched antibodies, B cells in germinal centers (GC) require cognate help from a subset of CD4 T cells called Tfh cells (78). Tfh cells express the chemokine receptor, CXCR5, which allows them to traffic to the B-cell zones of lymphoid tissue, thus, making them poised for proper T–B cell interactions. Without proper helper activity from Tfh, the antibody response is less robust, with lower titers, and exhibits reduced affinity maturation, which are two characteristics often associated with an aged antibody response (79).

CXCR5^+^ CD4 T cells, which resemble Tfh, are also found in human blood following vaccination (80). These peripheral blood dwelling Tfh, called circulating T follicular helper cells (cTfh), are CXCR5^+^PD-1^+^ but they differ somewhat in gene expression profiles from tissue Tfh. While they do not express the prototypical Tfh transcription factor Bcl-6, they can be identified as CD4^+^CXCR5^+^CXCR3^+^CCR6^−^PD-1^+^. Importantly, cTfh collected following vaccination correlate with production of a good humoral response and exhibit robust *in vitro* helper activity (81–83).

A recent study examined cTfh in young and older human subjects. It was found that there was a significant reduction of responding cTfh with age and that this was correlated with lower titers of IgG but not IgM production following vaccination. Aged cTfh also exhibited reduced *in vitro* helper activity when compared to young. In addition, aged cTfh examined post-vaccination exhibited lower levels of cell surface ICOS expression, which is critical for proper T–B cell interactions (84). Therefore, it is likely that these age-related defects in Tfh cells observed following vaccination contribute to the poor antibody response found in older individuals. Thus, a focus on strategies to enhance generation of Tfh is warranted.

### Suppressive Effects of Treg Cells are Enhanced with Aging

Regulatory T cells are defined by the expression of the forkhead/winged-helix family transcription factor FoxP3. Tregs can either be natural (nTregs), which are generated in the thymus, or induced (iTreg), which are generated in the periphery from non-Tregs following antigenic stimulation (85). Both of these Treg populations are capable of suppressing an immune response. The function of Tregs is useful to maintain peripheral tolerance but becomes problematic when it impacts an immune response to a vaccine or infection. One of the numerous changes in the immune system with aging is the accumulation of CD4^+^CD25^+^FoxP3^+^ Treg. Importantly, these Tregs can negatively impact a normal immune response, such as that to a tumor or infection (86, 87), in aging mice. Tregs from aged mice also produce higher levels of the inhibitory cytokine, IL-10, and suppress CD86 expression on DC more strongly than Tregs from young mice (88). Thus, not only are there more Tregs with aging, but they are also even better at suppressing an adaptive immune response. In order to improve vaccine efficacy for the elderly, this increase in Tregs needs to be taken into consideration. We have shown that when combined with influenza vaccine, a TLR4 agonist as a vaccine adjuvant improved the cell-mediated immune response in older adult PBMC through stimulation of inflammatory cytokines (IL-6 and TNFα) in myeloid DC, and was associated with a 10-fold reduction in IL-10 levels and an increase in GrzB produced by CD8 T cells in response to influenza challenge (89). Our future studies are focusing on the source of IL-10 production and how the T-cell response to influenza can be enhanced through the use of vaccine adjuvants.

### Improving the CD8 T-Cell Response to Influenza Vaccination

CD8^+^ CTLs are the dominant cytolytic effectors against influenza virus infection and are predominantly directed against the conserved internal proteins of the influenza virus, thus, conferring cross-protection against the different influenza A subtypes. However, these memory CTLs generated in response to prior influenza infections are poorly stimulated by inactivated influenza vaccines, particularly in older adults where age-related changes in CD8+ T cells correspond to increased risk of serious complications of influenza and a loss of vaccine effectiveness. Our research suggests that this age-related decline is reversible (67) and that vaccination strategies to improve antigen presentation of the internal proteins of the influenza virus including the use of vaccine adjuvants such as TLR ligands (89) may improve influenza vaccine-mediated protection in the older population (Figure 2).

### Understanding Inflammatory and Resiliency Factors to Improve Vaccine Responsiveness

Some of the same immune mediators, such as IL-6 that have been linked to inflammaging, frailty, and clinical outcomes, also play key roles in mediating and modulating immune responses to the influenza vaccine and infection. As a result, there is a natural tendency to explore ways in which specific categories of dysregulation of such molecules could play a shared role in all of these phenomena. One of the challenges in making such linkages stems from the fact that the use of immune or inflammatory molecules as biomarkers emphasizes their role as measurable predictors of clinically relevant events. Biomarkers have been defined as objectively measured subject characteristics that behave as indicators of normal biological processes, pathogenic mechanisms, or pharmacological responses to an intervention (90). In the context of inflammaging, biomarkers are generally those molecules (e.g., IL-6) that are sufficiently stable and abundant in peripheral blood samples to be easily measurable in frozen serum samples using standard techniques, such as enzyme-linked immunosorbent assays (ELISA), enabling their validation as predictors of relevant clinical outcomes (e.g., death, disability, frailty) in large epidemiologic studies.

Elevated plasma IL-6 levels do represent a validated predictor of declining mobility performance, disability, and death in older adults (91). Although knowledge of IL-6 biology does provide some broader insights into the implications of elevated peripheral blood IL-6 levels as regards pathogenic mechanisms, any direct translation is fraught with difficulties. Above all, IL-6 is a pleiotropic cytokine produced in T cells, macrophages, and non-immune cells (92). In blood, it crudely reflects synthesis in fat, liver, and muscle (92), yet locally, IL-6 levels are carefully regulated (92). Thus, significant differences may exist between IL-6 levels measured in peripheral venous blood and those observed within the tightly regulated local micro-environment of the “T-cell niche” within a specific tissue. Moreover, IL-6 may exert either pro- or anti-inflammatory effects depending on concentration, acuity of change, and presence of other modulators (92). For example, in the case of muscle, IL-6 promotes muscle catabolism (93) and insulin resistance (94) with chronic IL-6 administration inducing muscle atrophy (95). By contrast, IL-6 contributes to induction of skeletal muscle stem cell responses after exercise (96), while in its absence recovery from disuse atrophy (97) and overload-induced hypertrophy (98) are decreased.

Interventions designed to alleviate the negative effects of inflammaging on responses to influenza vaccine and infection will need to be carefully considered with all of the above considerations in mind. For example, the use of siltuximab, an anti-IL-6 monoclonal antibody (99), could at least in theory result in favorable effects by lowering the pro-inflammatory and catabolic effects of elevated IL-6 levels on muscle, while at the same time also negatively influencing the ability of muscle and T cells to respond to exercise and influenza vaccination/infection, respectively. By contrast, other interventions such as exercise (100), angiotensin receptor blockers such as losartan, and omega-3 polyunsaturated fatty acids (ω-3) such as fish oil may possibly exert more broadly favorable effects precisely by virtue of their highly pleiotropic effects. Inhibition of the mammalian target of rapamycin (mTOR) pathway extends lifespan in all species studied to date. A recent study has shown that the administration of rapamycin to older research volunteers improves many aspects of immunosenescence, including responses to influenza vaccination (101). These findings are in keeping with the Geroscience Hypothesis which postulates that the ability to target those specific pathways shared by aging and those chronic diseases for which aging is a predominant risk factor may help to prevent or delay the onset or progression of such diseases, thus, significantly expanding the human healthspan (102, 103).

## The Quest for T-Cell Correlates of Protection Against Influenza in Older Adults

Novel correlates of protection are needed to select and fast track the most promising new vaccines through the clinical development pipeline. Multiple components of immune function are affected during the aging process but few studies have included influenza surveillance to directly correlate these changes with influenza outcomes in the “usual” older adult population with multi-morbidity. There has been a paradigm shift in understanding the limitations of antibody titers as a sole measure of influenza vaccine efficacy in older people (48, 67, 68, 104) and although T-cell correlates of protection have been identified in young adults (63, 64), these correlates have yet to be translated to studies of older adults.

### Designing Studies to Develop Novel Correlates of Protection in Older Adults

Studies designed to establish new correlates of protection must prospectively enroll adequate numbers of older adults at the time of vaccination, and over multiple years due to highly variable attack rates, to detect enough influenza cases for a correlate of protection analysis. Since influenza A/H3N2 strains have the greatest impact in the over 65 population, this influenza subtype should be the primary focus for serologic and *ex vivo* T-cell assays of the response to vaccination. Key to the success of influenza surveillance and documentation of laboratory-confirmed influenza illness is recognizing the atypical presentation of influenza illness in older adults (105, 106), providing weekly reminder phone calls to study participants to report any acute respiratory symptoms, and offering the flexibility of study coordinators to procure nasopharyngeal (NP) swabs in the home or clinic within 5 days of the onset of illness (48, 67, 68). High-sensitivity, polymerase chain reaction (PCR)-based assays are required to reliably detect influenza virus in the NP swab specimens. Antibody seroconversion in response to an influenza illness is unreliable in this population; we have shown that older persons with febrile influenza illness may be PCR+ for influenza virus but are unable to mount an antibody response to influenza infection even though their antibody responses to vaccination were similar to other older adults who do not develop influenza illness (68). Studies of the immune response pre- and post-infection may also offer insights into potential correlates of protection as infection provides a more robust stimulus to the immune system compared to inactivated influenza vaccines.

### Summary

The age-related loss of vaccine-mediated protection against influenza goes beyond that which can be explained by immunosenescence. Multi-morbidity, frailty, and functional dependence accelerate changes in a number of body systems, including mucosal barriers and innate and adaptive immune functions. The increased risk for serious complications of influenza infection despite widespread vaccination programs is a major public health concern. The loss of antibody-mediated protection against influenza with age and over-reliance on this correlate of protection in the vaccine development pipeline has posed significant challenges to improve protection against influenza in the over 65 population. The interactions of immune senescence, persistent CMV infection, inflammaging, and dysregulated cytokine production are only beginning to be understood and appear to have a significant impact on T-cell-mediated immunity needed for clinical protection against serious influenza illness. Developing new vaccine formulations and routes of administration and establishing novel correlates of protection against influenza that predict improved outcomes in older adults are key to expediting the process for new vaccine development and reducing the risk of failure in late phase clinical trials.

## Author Contributions

JM organized the different sections of the manuscript, consulted with other authors on sections for their contributions, assembled the draft sections, sent out for review to all authors, consolidated all of the edits, and finalized the manuscript for submission. GK wrote the section on inflammation and interactions between immune senescence and common pathways for age-related changes. XZ wrote draft sections and provided to JM, on age-related changes in the human CD8+ T cell response to influenza. SS wrote several sections related to age-related changes in adaptive immune mechanisms in the mouse model and potential for translation to human. LH wrote the sections on T follicular and T regulatory cells and how experiments in mice can be translated to studies in human peripheral blood.

## Conflict of Interest Statement

JM has participated on advisory boards for GlaxoSmithKline, Sanofi Pasteur, and Pfizer, and on data monitoring boards for Sanofi Pasteur; she has received research grants from the Canadian Institutes of Health Research and the US National Institute of Allergy and Infectious Diseases, has participated in clinical trials sponsored by Merck, GlaxoSmithKline, and Sanofi Pasteur, has received honoraria and travel and accommodation reimbursements for presentations sponsored by GlaxoSmithKline, Merck, and Sanofi Pasteur, and travel reimbursement for participation on a publication steering committee for GlaxoSmithKline. GK, XZ, SS, and LH have no conflicts to declare.
